# Supplementary material for: Dislocation interactions during plastic relaxation of epitaxial colloidal crystals
Source: Nat Commun. 2023 Sep 16;14:5760. doi: 10.1038/s41467-023-41430-3 (PMC10505195; doi:10.1038/s41467-023-41430-3)
Supplement: Supplementary file 1 — Supplementary Information [file 41467_2023_41430_MOESM1_ESM.pdf]

Supplementary Information for Dislocation interactions during plastic relaxation of epitaxial colloidal crystals

Ilya Svetlizky,[1,*] Seongsoo Kim,[1,*] David A. Weitz,[1,2,3] and Frans Spaepen[1]

[1]*School of Engineering and Applied Sciences (SEAS), Harvard University,Cambridge, MA, USA*

[2]*Department of Physics, Harvard University, Cambridge, MA, USA*

[3]*Wyss Institute for Biologically Inspired Engineering, Harvard University, Cambridge, MA, USA*

[*] These authors contributed equally to this work

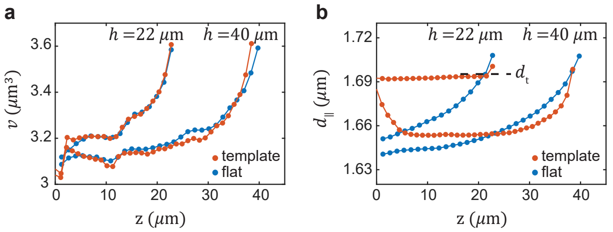

Supplementary Figure 1. Comparison between the growth of a crystal on a template (constrained) and on a flat substrate (unconstrained), at different heights, h. Profiles of the volume per particle $v(z)$ (a), and in-plane particle-particle nearest neighbor distance $d_{\|}(z)$ (b), for constrained (orange) and unconstrained (blue) crystals. The examples are chosen to reflect the profiles below ($h = 22\ \mu$m) and above ($h = 40\ \mu$m) the critical height, and correspond to the experiments presented in Fig. 1d of the main text. Here, the ionic strength $I = 2$ mM and template spacing $d_{\mathrm{t}} = 1.69(5)\ \mu$m.

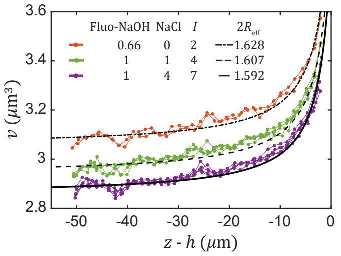

Supplementary Figure 2. Sedimentation of nearly-hard-sphere colloids on a flat substrate. $v(z)$ profiles measured for $h = 50\ \mu$m thick crystals in solutions of different levels of ionic strength, I. $z - h = 0$ denotes the crystal-fluid interface. Measured profiles are approximated well by the hard-sphere model (Eqs. 3 & 4), denoted by the solid and dashed black lines. (legend) Concentrations of the dye, NaCl, and the ionic strength are given in mM. The effective particle diameters $2R_{\mathrm{eff}}$ used in the hard-sphere model are specified in μm. Plotted profiles correspond to the experiments used in Figs. 2, 4 and 5 of the main text.

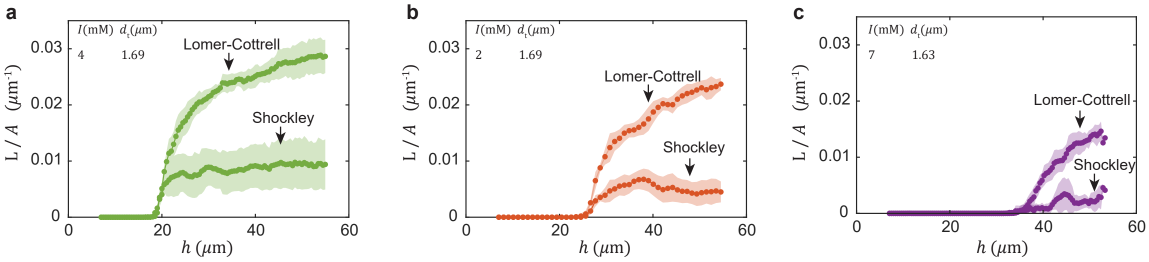

Supplementary Figure 3. Comparison between growth of Lomer-Cottrell and Shockley dislocation areal densities. (**a-c**) Evolution of areal dislocation densities, L/A, during crystal growth. Lomer-Cottrell and Shockley type dislocations are plotted separately. After initial rapid growth of the networks, Lomer-Cottrell dislocations constitute $\sim 80\%$ of the network length. Plotted dislocation densities correspond to the experiments used in Figs. 2, 4 and 5 of the main text.

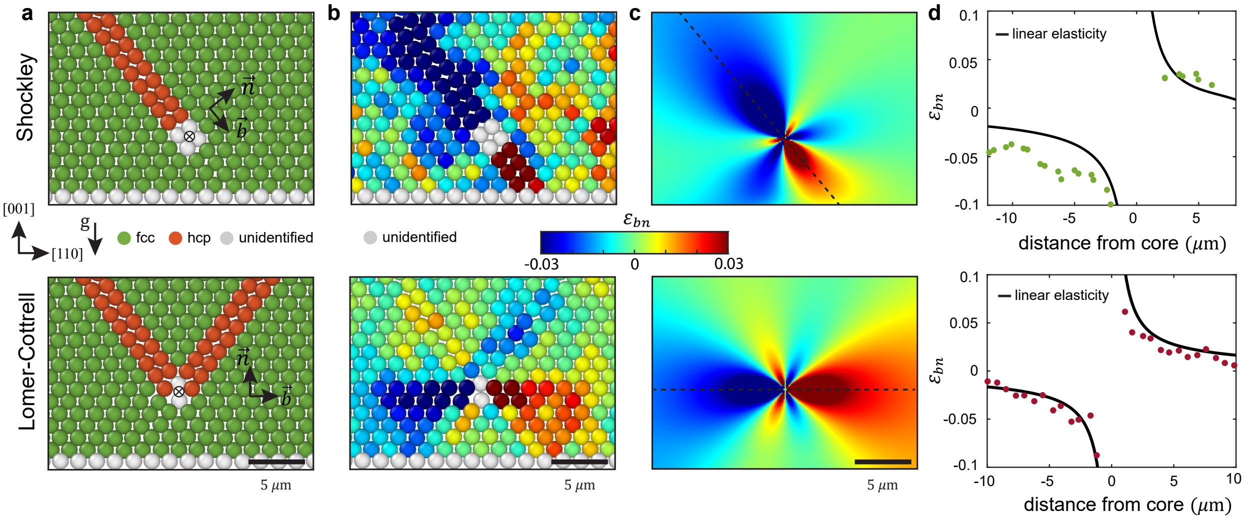

Supplementary Figure 4. Crystalline structure and elastic strains near Shockley (top row) and Lomer-Cottrell (bottom row) edge dislocations. (**a**) The dislocation lines run perpendicular to the page and marked by $\otimes$; they bound hcp (orange) stacking faults in fcc (green) crystals. The crystalline structure is unidentified (white particles) in the close vicinity of the dislocation lines. The layer of the unidentified particles at the bottom marks the first layer of particles above the rigid template, which establishes zero displacement boundary conditions. (**b**) Color map of the resolved elastic strains ε_{bn}, where $\mathbf{n}$ is the normal to the plane defined by the Burgers vector $\mathbf{b}$ and the dislocation line, as shown in (**a**). We subtract the value of ε_{bn} that corresponds to the average misfit strain. (**a**, **b**) The particle positions were averaged over 15 layers along the dislocation line ([1$\bar{1}$0] direction). (**c**) Linear elastic prediction of ε_{bn} for dislocations near an infinitely stiff substrate (see Modeling strain relaxation section in Methods), with no adjustable parameters. (**d**) The measured ε_{bn} (solid points) and the analytic solution (black lines) are compared along the dashed lines in panel ()**c**).

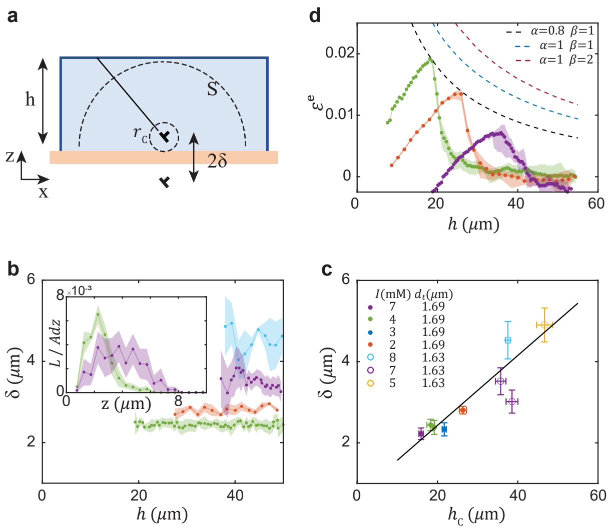

Supplementary Figure 5. Dislocation-substrate seperation distance and the relaxation model. (**a**) Schematic of the model. The edge dislocation lies along the y axis at a distance δ above the stiff substrate. An image dislocation of the same Burgers vector at a distance δ below the substrate reflects the repulsion from the substrate. The Energy functional (Eq. 5) is integrated over area S, with an outer and inner radii, h (crystal thickness) and r_{c} (dislocation core radius), respectively. (**b**, inset) Examples of measured z-profiles of dislocation densities calculated over thin slices of volume $A \times dz$. (**b**, main) Average values of δ calculated from the density profiles (inset) and plotted as a function of h. Whereas δ does not change during the crystal growth, it varies with the mismatch level. Colors correspond to experiments with different values of I and d_{t}, as indicated in panel (**c**). (**c**) For each experiment, the average δ is plotted with respect to the critical thickness, h_{c}. A linear fit, black line, is used in the evaluation of $\mathcal{E}_{\mathrm{L}}(x, z; \delta(h), \mu(z), \nu)$. (**d**) Comparison between the experiments and the relaxation model for Shockley partial dislocations for different values of $\beta = b/r_{\mathrm{c}}$ and the adjustable parameter α (see text).
