# Supplementary material for: Dislocation interactions during plastic relaxation of epitaxial colloidal crystals
Source: Nat Commun. 2023 Sep 16;14:5760. doi: 10.1038/s41467-023-41430-3 (PMC10505195; doi:10.1038/s41467-023-41430-3)
Supplement: Supplementary file 3 — Description of Additional Supplementary Files [file 41467_2023_41430_MOESM3_ESM.pdf]

Description of Additional Supplementary Files

Supplementary Movie 1 Growth of a colloidal polycrystal

Confocal microscopy is used to visualize the growth of a colloidal crystal on a flat substrate. The time series demonstrates the sedimentation process and growth of columnar fcc (green particles) grains with hcp (orange particles) stacking faults. Grain boundaries and the sedimenting particles above the crystal are not identified with a crystalline structure and are marked by gray particles. Only a small portion of the crystal is presented.

Supplementary Movie 2 Growth of a colloidal single crystal

Confocal microscopy is used to visualize the growth of a colloidal crystal on a templated substrate with a square pattern. The template constrains the first layer of particles and dictates the growth of a single fcc crystal (green particles). The crystal's growth is unstable: as the crystal reaches a critical height, multiple hcp (orange particles) stacking faults are formed, a process that relaxes the misfit strain imposed by the template.

Supplementary Movie 3 Evolution of the dislocation network - low mismatch

Time series of a growing misfit dislocation network (top view) in a crystal grown on a low mismatch template (purple data set in Figs. 2,4,5). The network consists of two perpendicular sets of parallel lines along the two principal directions of the template, [110] and [1-10], and is obtained by excluding the thread part of dislocations, which extends across the crystal height. The formed network is characterized by well-ordered arrays of long dislocation segments of predominantly Lomer-Cottrell type (red segments).

Supplementary Movie 4 Evolution of the dislocation network - high mismatch

Time series of a growing misfit dislocation network (top view) in a crystal grown on a high mismatch template (green data set in Figs. 2,4,5). The network consists of two perpendicular sets of parallel lines along the two principal directions of the template, [110] and [1-10], and is obtained by excluding the thread part of dislocations, which extends across the crystal height. During the early stages of the relaxation process, the formed network is characterized by a very fragmented structure that consists of short dislocation segments of predominantly Lomer-Cottrell type (red segments). The growth of the segments is blocked either by the formation of Hirth immobile junctions (yellow segments) or by the presence of perpendicular segments. As the crystal grows thicker, dislocations overcome these blocking mechanisms, forming an elongated array of dislocations.
